# Hot Springs. Ethics or Quackery, Which?

**Published:** 1878-04

**Authors:** Charles A. Wall

**Affiliations:** Buffalo


### Correspondence.

Buffalo, March 12th, 1878.

MESSRS. EDITORS.*

I would call your attention to the state of the profession in Arkansas, especially in reference to the "Hot Springs Illustrated Monthly," a secular newspaper published at Hot Springs, Ark., and its list of advertised physicians. It contains the pictures of Drs. G. W. Lawrence, P. H. Ellsworth and O. A. Hobson, with brief biographies of each. It is only necessary for us to refer medical men to the code of ethics, for them to determine at once the position that these men occupy. Dr. McClelland, in *Civil Malpractice* in defining what constitutes a 'quack,' says, "This term, in the medical profession, is applied to any one, whether he has any professional education or not, who violates §§ 3 and 4, Art. I, ch. II, of what is known as the Code of Ethics of the American Medical Association, which declares it to be derogatory to the dignity of the profession to resort to public advertisements, or private cards, or hand bills, inviting the attention of individuals affected with particular diseases."

If allowing portraits and biographies of physicians to be placed in a secular paper does not constitute a violation of the section

* The above was handed to us by our young friend and we publish it without comment, as it may be of interest to many readers. There seems to be room for reform at the Hot Springs. ED.

quoted, we are at a loss what conclusion to draw in these cases. Drs. Ellsworth and Hobson are both members of the county medical society, which, after hearing and considering charges made against them, has declared them not guilty. This decision is beyond us, but as we hear that a second number containing the photographic likenesses of three other physicians* with accompanying biographies has been issued, perhaps it may be accounted for, by supposing a majority of the doctors composing the society, to be ambitious to have their faces engraved and circulated broadcast over the country. We may be severe, but from the premises what other conclusion can follow.

We hope the State Society of Arkansas will, at its earliest convenience, take cognizance of this breach of etiquette and punish the violators as they deserve. If they do not we shall look to the American Medical Association as the last resort. If the State Society and the American Medical Association do not see fit to condemn the actions of these bidders for public patronage, then there can be but one result, the universal repudiation of the Code of Ethics as unjust and discriminating. Why the action of the Medical Society, of the County of New York, directing the withdrawal of the names of its members from the recommendations of remedies announced in medical journals, if their brethren (?) in Arkansas are allowed to advertise themselves in the secular press circulated among the people, from whom they draw their patronage?

We are not to be understood to insinuate that all the physicians in Hot Springs are longing for 'forbidden fruit,' far from it, as the following from the "Illustrated" will show: "We are sorry to learn that some of our physicians have objected to these illustrations, so far as the doctors are concerned, as being unprofessional and in bad taste." The editors of the monthly then goes on to say that they differ, and that it is an *honor* not sought by the subjects but is bestowed unasked "*after first ascertaining, of course, that such publications will not be offensive to the parties.*" The sketches, it seems, are not, as we at first supposed, autobiographies, but the physicians furnish their photographs and the facts in their

*Since writing the above I have ascertained that the doctors advertised in the second number are, S. W. Franklin, S. Vaughan and E. A. Shippey, all of Hot Springs. I am unacquainted with the medical relations of these men since I have not seen their biographies. C. A. W.

lives, while the paper furnishes the woodcuts and arranges the facts. Deliver us from our friends if we must be thus *honored* at the expense of our manhood. Hoping that the medical profession will take this up and bring the culprits before the bar of professional justice I remain Messrs. Editors, Yours,

CHARLES A. WALL.

